# T247. INSIGHT INTO NEGATIVE SYMPTOMS AS AN IMPORTANT TARGET FOR PSYCHOSOCIAL REHABILITATION IN RELATION TO CLINICAL CHARACTERISTICS

**DOI:** 10.1093/schbul/sby016.523

**Published:** 2018-04-01

**Authors:** Maria Minyaycheva, Igor Gladyshev, Oleg Papsuev

**Affiliations:** Moscow Research Institute of Psychiatry

## Abstract

**Background:**

Apathy and amotivation are considered as the core features of negative symptoms in patients with schizophrenia spectrum disorders. It’s well know that schizophrenia patients often lack insight into their symptoms. Insight bias affects self-representation, social functioning and social outcomes, reduces effects of psychosocial treatment and rehabilitation.

**Objective:**

To research key aspects of insight into apathy depending on diagnostic categories in patients with schizophrenia spectrum disorders. The aim of the study was to analyze correlations of insight into apathy/amotivation with clinical symptoms, compliance with treatment and social cognition.

**Methods:**

103 patients with schizophrenia and schizophrenia spectrum disorders were recruited to participate in the study. Only patients in stabilized state that met criteria of PANSS total score ≤ 80 points were included. Demographic data was collected along with the clinical description on prevailing symptoms during acute phase. Discrepancy score for Apathy Evaluation Scale clinical (AES-C) and self-rated (AES-S) versions was used to assess insight into amotivation syndrome. Hinting Task, Ekman-60 and RAD-15 were used to assess social cognition and BACS was used for neurocognition.

**Results:**

Overall, moderate positive correlations between AES-C and PANSS amotivation subscale N2 and N4 items, N6 item with total PANSS negative subscale were revealed. No significant correlations with G16 item were registered. AES-C/AES-S discrepancy ratio also modestly correlated with paranoid schizophrenia (r=0,29) and prevailing delusional symptoms during acute phase (r=0,33) of manifest psychoses, age of onset (r=0,28) and inpatient only treatment intake (r=0,27). It was negatively correlated with number of hospital admissions (r=-0,43). It is worth noting that we found no correlation between AES discrepancy ratio and social cognition and neurocognition.

**Discussion:**

Patients with prevailing paranoid symptoms not only lack insight into positive symptoms, but tend to underestimate their negative symptoms such as motivation and apathy. Clinically this can be described by overestimated strengths, overstated expectations, exaggerated hopes, mistakenly overrated beliefs. These phenomena often biases the recovery process and need to be addressed during motivational enhancement therapy. Patients with more difference between the results in AES-C and AES-S are less critical to their conditions and less committed to therapy while being more paranoid in their beliefs. It is also harder to identify problems and targets for these patients as they often see no reasons for treatment at all. Probably with some of these patients indirect methods (metacognitive training) would be preferable rather than psychoeducation-based approaches when choosing psychological therapies. Interestingly no relationship of insight and social cognition was revealed. That needs further investigation as motivation is often considered to be a mediator for neurocognitive and social cognitive functions while there is still little works on the role of insight in relation to social cognition.

